# Unraveling the Biology of Epithelioid Hemangioendothelioma, a TAZ–CAMTA1 Fusion Driven Sarcoma

**DOI:** 10.3390/cancers14122980

**Published:** 2022-06-16

**Authors:** Caleb N. Seavey, Ajaybabu V. Pobbati, Brian P. Rubin

**Affiliations:** 1 Department of Cancer Biology, Lerner Research Institute, Cleveland Clinic Foundation, Cleveland, OH 44195, USA; seaveyc@ccf.org (C.N.S.); pobbata@ccf.org (A.V.P.); 2 Department of General Surgery, Digestive Disease and Surgery Institute, Cleveland Clinic Foundation, Cleveland, OH 44195, USA; 3 Department of Molecular Medicine, Cleveland Clinic Lerner College of Medicine, Case Western Reserve University, Cleveland, OH 44195, USA; 4 Robert J. Tomsich Pathology and Laboratory Medicine Institute, Cleveland Clinic Foundation, Cleveland, OH 44195, USA

**Keywords:** YAP/TAZ, hippo pathway, epithelioid hemangioendothelioma, TAZ–CAMTA1, sarcoma

## Abstract

**Simple Summary:**

Epithelioid hemangioendothelioma (EHE) is a rare vascular cancer that involves a gain-of-function gene fusion involving TAZ, a transcriptional coactivator, and one of two end effectors of the Hippo pathway. Although the activity of TAZ and/or YAP, a paralog of TAZ, is consistently altered in many cancers, genetic alterations involving YAP/TAZ are rare, and the precise mechanisms by which YAP/TAZ are activated are not well understood in most cancers. Because *WWTR1(TAZ)–CAMTA1* is the only genetic alteration in approximately half of EHE, EHE is a genetically clean and homogenous system for understanding how the dysregulation of TAZ promotes tumorigenesis. Therefore, by using EHE as a model system, we hope to elucidate the essential biological pathways mediated by TAZ and identify mechanisms to target them. The findings of EHE research can be applied to other cancers that are addicted to high YAP/TAZ activity.

**Abstract:**

The activities of YAP and TAZ, the end effectors of the Hippo pathway, are consistently altered in cancer, and this dysregulation drives aggressive tumor phenotypes. While the actions of these two proteins aid in tumorigenesis in the majority of cancers, the dysregulation of these proteins is rarely sufficient for initial tumor development. Herein, we present a unique TAZ-driven cancer, epithelioid hemangioendothelioma (EHE), which harbors a *WWTR1(TAZ)–CAMTA1* gene fusion in at least 90% of cases. Recent investigations have elucidated the mechanisms by which YAP/TAP-fusion oncoproteins function and drive tumorigenesis. This review presents a critical evaluation of this recent work, with a particular focus on how the oncoproteins alter the normal activity of TAZ and YAP, and, concurrently, we generate a framework for how we can target the gene fusions in patients. Since EHE represents a paradigm of YAP/TAZ dysregulation in cancer, targeted therapies for EHE may also be effective against other YAP/TAZ-dependent cancers.

## 1. Introduction

The dysregulation of mitogenic and/or growth-suppressor signaling cascades is a hallmark of oncogenesis [1]. One such frequently altered growth-suppressor cascade is the Hippo pathway, and several studies have shown that the derangement of this pathway across all solid cancers is a major mechanism of tumorigenesis [2,3].

**Figure 1:**
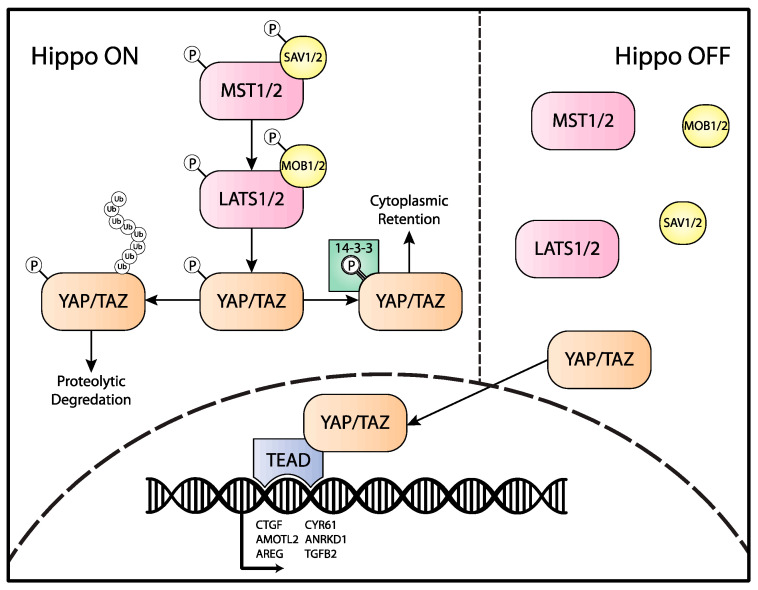
Schematic of the Hippo pathway. The top left represents the activation of the Hippo pathway with phosphorylated core Hippo kinases and YAP/TAZ. Phosphorylated YAP/TAZ leads to cytoplasmic retention via binding to 14-3-3 proteins and/or polyubiquitination and proteolytic degradation. Upon Hippo inactivation (top right), YAP/TAZ can migrate to the nucleus, where it can bind to its TEAD cofactors and activate transcription.

Two serine–threonine kinases and two adaptor proteins constitute the core components of this pathway [4,5,6,7,8,9]. The activity of the core components is tightly regulated, as they serve as a platform to integrate signals from other pathways to yield downstream transcriptional alterations [10,11]. The regulatory processes that feed into this pathway include biomechanical cues, GPCR signaling, and cell-to-cell contact signaling [12,13,14,15,16,17,18,19,20,21,22,23,24]. The serine–threonine kinases of this pathway include Mammalian Sterile 20-related 1 and 2 kinases (MST1 and MST2) and Large Tumor Suppressor 1 and 2 kinases (LATS1 and LATS2) [4,5,9]. MST1 and -2 are activated through homodimerization and trans-autophosphorylation, and these processes are facilitated by the adaptor protein SAV1 (Figure 1) [7,8,25,26,27]. Then, MST1/2 phosphorylates LATS1 and LATS2, as well as their adaptors, Mps One Binder 1A and 1 B (MOB1A/B) [27,28,29,30]. Phosphorylation enhances the interaction between LATS1/2 and MOB1A/MOB1B proteins and leads to the activation of the complex [28,31]. Activated LATS/MOB1 then inhibits the end-effector transcriptional coactivators Yes-Associated Protein 1 (YAP1 (also known as YAP)) and transcriptional coactivator with PDZ-binding motif (TAZ) via phosphorylation [21,32,33]. LATS1/2 are serine–threonine kinases that phosphorylate YAP at positions S61, 109, S127, S164, and S381, and TAZ at positions S66, S89, S117, and S311. The phosphorylation of these residues leads to either cytoplasmic retention via binding to 14-3-3 proteins or ubiquitin-mediated proteolytic degradation [21,33,34]. The phosphorylation of these two proteins is a critical step that yields the overall effect on the Hippo pathway. Therefore, in a “Hippo-On” state, the activation of the Hippo pathway yields the inactivation of YAP/TAZ (Figure 1).

In comparison with the “Hippo-On” state, in a “Hippo-Off” state, inactive Hippo-pathway kinases yield unphosphorylated YAP/TAZ, which are active and therefore able to translocate to the nucleus. As neither YAP nor TAZ contain DNA-binding domains, these transcriptional coactivators require TEA domain 1–4 (TEAD1-4) transcription factors [35,36,37,38]. In effect, the TEAD1-4 protein tethers YAP/TAZ to genic loci and alters transcriptional activity. Although the targets of YAP/TAZ–TEAD are context-specific, the core conserved transcriptional targets include mediators that enhance proliferation, survival, and cell migration [10,33,35,38,39,40].

Paradoxically, although the activity of YAP/TAZ in cancer is most commonly pro-tumorigenic, in certain cancers, YAP/TAZ activity can be antitumorigenic [41,42,43]. Furthermore, the degree of “oncogenic addiction” to YAP/TAZ varies among cancers [44]. Therefore, as the therapeutic inhibition of YAP/TAZ is currently being evaluated in phase 1 clinical trials that target TEAD, identifying cancers that display the greatest reliance on YAP/TAZ activity is essential for credentialing TEAD inhibitors [45]. In this space, epithelioid hemangioendothelioma (EHE) is an excellent model cancer, as it is a monogenic neoplasm that is addicted to a fusion gene that functions as a constitutively active TAZ.

## 2. Epithelioid Hemangioendothelioma

Epithelioid hemangioendothelioma (EHE) is a rare vascular sarcoma that was originally described by Weiss and Enzinger in 1982 as a soft-tissue tumor with both a vascular morphology and a borderline histologic phenotype, with both benign and malignant features [46]. Clinically, EHE is most commonly diagnosed in patients in their mid-30s (range: 8–90s) [47,48]. While this disease was initially described in soft tissue and bone, it is now realized that it most commonly occurs within the liver, followed by the lungs and bones/soft tissue [47,49]. The clinical course of this disease is diverse, ranging from prolonged stability and long-term survival to rapidly progressive disease with high morbidity and mortality [48,50,51].

Attempts have been made to stratify EHE into either aggressive or indolent diseases [47,48,50]. In EHE, the anatomical location of the tumor has significant prognostic value [48,50,51]. Patients with isolated soft-tissue disease have the best overall survival (87% 5-year survival), with liver and lung involvement demonstrating worse survival (65% and 45% 5-year survival, respectively) [47]. Furthermore, while uncommon, patients with peritoneal- and pleural-surface involvement have a moribund prognosis [47,48,50,51]. While the site of disease correlates with the overall prognosis, there is still a large variability in survival among patients.

Histologically, EHE is characterized by the presence of cords or nests of epithelioid cells, which are cells that contain a rounded cell body [46,50,52]. The rounded-cell-body structure of epithelioid cells contrasts with the elongated spindle shape of spindle cells. These tumor cells are typically set in a dense myxohyaline stroma and often contain intracytoplasmic vacuoles [46,50,52]. Importantly, subtle differences in the histological morphology are essential for the subclassification of endothelial-cell tumors. Angiosarcoma, the most common vascular sarcoma, contains primitive and poorly organized vascular structures that are lined by atypical endothelial cells [53]. In contrast, EHE does not contain these “vasogenic features” and it can be differentiated by H&E staining by the presence of the characteristic and abundant myxohyaline stroma, in which the tumor cells are embedded.

Further highlighting its endothelial lineage, EHE expresses the endothelial-cell markers PECAM1, CD34, and ERG [50,53]. For a significant proportion of tumors, no originating vessels are identified [46,52,54]. However, for tumors that have a visible originating vessel, the tumor typically displays an angiocentric growth pattern, rather than intraluminal growth, with the tumor bulk growing into the surrounding structures adjacent to the vessel [53,54]. Often, the vessel will eventually thrombose and/or be obliterated as the tumor encases it.

## 3. Molecular Alterations in EHE

**Figure 2:**
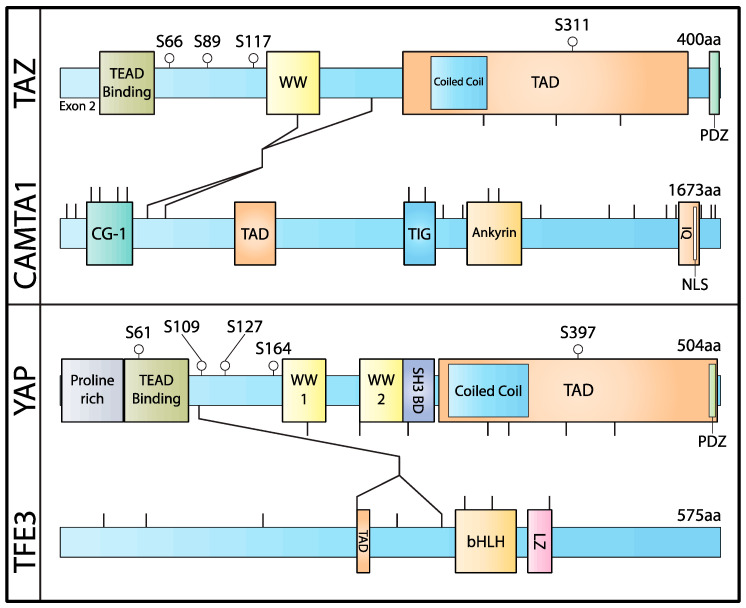
Schematic of the proteins present in TAZ–CAMTA1 and YAP–TFE3 fusions, with the most common breakpoints within the proteins. TAZ and YAP are labeled above with the LATS1/2 phosphorylation sites. The markers below display the amino acid contributions of each exon, and the lines between sequences denote common fusion sites: WW: WW domain; TAD: transactivation domain; PDZ: PDZ-binding motif; CG-1: CG-1 DNA-binding domain; TIG: transcription-factor immunoglobulin domain; Ankyrin: ankyrin repeats; IQ: IQ calmodulin-binding motifs; NLS: nuclear-localization signal; SH3 BD: SH3-binding domain; bHLH: basic helix–loop–helix domain; LZ: leucine-zipper domain.

In 2011, two significant publications independently identified a fusion gene that defined EHE. Although it had previously been demonstrated that EHE samples contained a chromosomal 1,3 reciprocal translocation t (1p36;3q25), which was observed by karyotyping, the resultant fusion gene was unknown [55]. Tanas et al. performed whole-transcriptome sequencing with a fusion-detection algorithm and identified the *WWTR1(TAZ)–CAMTA1* (WW-Domain-Containing Transcription Regulator 1–Calmodulin-Binding Transcription Activator 1) gene fusion [56]. This publication further demonstrated that this alteration is present in approximately 90% of clinical samples of EHE and is not identified in any other vascular tumor. These results were corroborated by the findings of Errani et al., who similarly identified the *WWTR1(TAZ)–CAMTA1* gene fusion by FISH positional cloning, followed by RT-PCR [57]. These two studies demonstrated that this gene fusion is a defining feature of the disease; therefore, the detection of the *WWTR1(TAZ)–CAMTA1* alteration is now commonly used in the histopathologic workup for classifying tumors as EHE [53,58]. In EHE, the most common fusions identified were those joining exon 3 of *WWTR1* with exons 8 or 9 of *CAMTA1* (Figure 2) [56,57]. However, the entire TEAD-binding motif is always present, suggesting that the interaction with TEAD is important for oncogenesis.

In addition to the *WWTR1(TAZ)–CAMTA1* fusion characteristic of EHE, a *YAP1–TFE3* fusion was identified in a related lesion that we now refer to as *YAP1–TFE3*-rearranged hemangioendothelioma [59,60]. The first *YAP1–TFE3* fusion was generated by joining the first exon of *YAP1* to the fourth exon of *TFE3,* which yielded an in-frame transcript (Figure 2). *YAP1–TFE3*-fused HE displays a unique histological identity when compared with *WWTR1–CAMTA1*-fused EHE. Indeed, this novel subtype of HE shows more variable histological features, with the common feature of large eosinophilic cells, most often arranged in large nests, and lacking myxohyaline stroma. In addition to the initially described *YAP1* exon 1–*TFE3* exon 4 fusion, a further *YAP1* exon 1–exon 6 fusion was identified (Figure 2) [61]. Recently, we presented the largest case series of *YAP1–TFE3*-fused HE, of which the majority contained a *YAP1* exon 1–*TFE3* exon 4 fusion (88%), and a minority contained a *YAP1* exon 1–*TFE3* exon 6 fusion (12%) [60]. When compared with the behavior of *WWTR1–CAMTA1* EHE, patients harboring *YAP1–TFE3*-fused HE were more likely to have soft-tissue disease and had an improved 5-year progression-free survival [47,50]. Since the initial discovery of this fusion, the *YAP1–TFE3* fusion has also been identified in the clear-cell stromal tumor of the lung and in a case of cutaneous low-grade fibromyxoid neoplasm; no additional cancers have been identified that harbor the *WWTR1–CAMTA1* fusion [62,63,64].

While the *WWTR1–CAMTA1* fusion is present in nearly all clinical samples of EHE, much rarer fusions are still being identified. A case series of six samples of EHE that did not contain the *WWTR1–CAMTA1* fusion identified two further alterations involving *WWTR1*: the *WWTR1–MAML2* and *WWTR1–ACTL6A* fusions [65]. Furthermore, they identified three clinical samples that contained a *WWTR1* fusion, but no 3′ partner was identified. Overall, this cohort of variant *WWTR1*-fused EHE had a large predilection for development in the heart muscle, which, altogether, is a rare location for the development of EHE. It is uncertain if these alternative fusions alter the clinical course of patients with EHE, and with the continued use of NGS in the diagnosis of EHE, it is likely that additional rare fusions will be identified in EHE.

While there has been significant success in the identification of driver fusions within EHE, limited efforts have been made to identify secondary mutations, which may alter the disease phenotype. Seligson et al. specifically aimed to identify these secondary mutations and correlate them with clinical characteristics [66]. Concurrent with our previous findings, they demonstrated that approximately 90% of the clinical samples of EHE in their large cohort contained the *WWTR1–CAMTA1* fusion. They further found that 57% of the EHE samples contained secondary mutations and, most commonly, alterations in *CDKN2A/CDKN2B*. Furthermore, in their cohort, patients with secondary mutations were more likely to have later-stage disease (stages III and IV). While this work has broadened our understanding of the mutational landscape of EHE, it is uncertain whether these secondary mutations mechanistically enhance tumorigenesis in a substantial way or whether their presence is solely a marker of late-stage disease. Nevertheless, this investigation has enhanced our view of the genetic alterations within this disease, and further clinical and mechanistic research is necessary to identify whether these mutations are associated with survival differences or altered treatment responsiveness.

## 4. The Biology of *WWTR1*(TAZ)–*CAMTA1* Fusion

Following our discovery of the *WWTR1–CAMTA1* gene fusion in EHE, we aimed to identify the mechanisms that underly tumorigenesis. *WWTR1*, which codes for the TAZ protein, is a transcriptional coactivator and end effector of the Hippo pathway [33,35]. In comparison, CAMTA1 is an incompletely understood protein. The protein belongs to a family of calmodulin-binding activators and it acts in calcium signaling, neuronal processes, and most notably, in memory [67,68]. CAMTA1 is suspected to be a tumor suppressor [69,70,71,72,73,74]. In this work, we demonstrated that the TAZ–CAMTA (TC) fusion protein functions as an activated TAZ-like protein and can induce cell transformation when transfected into NIH3T3 cells [75]. In contrast, full-length TAZ, full-length CAMTA1, and truncated TAZ and CAMTA1 portions of TAZ–CAMTA1 were not sufficient to induce cell transformation, thereby demonstrating that both the N- and C-terminal portions of the protein are necessary for TAZ–CAMTA1′s function. At its N-terminus, TC possesses a TEAD-interacting motif that interacts with the TEAD family of transcription factors (Figure 2). The introduction of a single point mutation in TC that abrogates TEAD binding abolishes the ability of TC to induce soft-agar colony growth. Therefore, TEAD binding is critical for transformation. Mechanistically, we identified a C-terminal nuclear-localization sequence (NLS) within the CAMTA1 protein and demonstrated that this motif is necessary for the tumorigenic capacity of the protein (Figure 2). Because the activity of YAP/TAZ is primarily regulated by its subcellular localization, an NLS from CAMTA1 localizes the fusion protein to the nucleus, thereby enhancing its function. 

## 5. Understanding the YAP1–TFE3 Fusion

While there is a significant overlap between the biology of this YT fusion and the TC and YAP fusions that are seen in other cancers, two recent studies have shown that YT displays unique features that distinguish it from other YAP/TAZ fusions.

All the YT fusions identified thus far either joined the first exon of *YAP1* to the fourth exon of *TFE3*, or the first exon of *YAP1* to the sixth exon of *TFE3.* However, this N-terminal portion of YAP still retains the TEAD-binding motif, and, thus, YT fusions can associate with the TEAD family of transcription factors (Figure 2) [76,77]. Furthermore, similar to TAZ, YAP contains five key serine residues that are used by the Hippo pathway to regulate YAP activity (Figure 2) [32,33,38]. Analogous to the S89 residue in TAZ, the S127 residue in YAP, when phosphorylated, facilitates 14-3-3 binding and cytoplasmic retention. The phosphorylation of the other four serine residues (residues other than S127) also leads to an inhibitory effect, albeit to a lesser degree [78]. In comparison to full-length YAP, the YT fusion contains only one of these five serine residues, and, importantly, S127 is not present in the fusion [76,77]. In contrast, TC contains three of the four regulatory serine sites in TAZ and contains the highly regulated S89 residue [75]. Therefore, YT appears to be insensitive to Hippo-pathway activity [76,77].

Two recent studies have broadened our understanding of the mechanisms driving YT-mediated tumorigenesis. Szulzewsky et al. aimed to identify the mechanisms that drive tumorigenesis in multiple YAP fusions, including *YAP1–TFE3* (*YAP1–TFE3*-fused hemangioendothelioma), *YAP1–MAMLD1* (supratentorial ependymoma), *YAP1–FAM118B* (supratentorial ependymoma), and *YAP1–SS18* (cervical squamous cell carcinoma and endocervical adenocarcinoma) [76]. In this work, the authors used heterologous expression systems to demonstrate the tumorigenic capacity for these fusions, and to elucidate the mechanisms by which cellular transformation occurred. Each fusion was sufficient to induce tumorigenicity when expressed within cells that express the tv-a receptor. Similar to our findings with TC, all four YAP fusions induced a YAP/TAZ–TEAD-enriched transcriptional profile. Furthermore, this study identified nuclear-localization sequences within the fusion partners that drive the constitutive nuclear localization of these proteins. Finally, while the wild-type YAP is degraded and/or retained in the cytoplasm in Hippo-pathway-activated states, the fusion proteins were resistant to degradation and remained nuclear-localized in such states, thus demonstrating that these fusions are dissociated from the upstream regulation of the Hippo pathway [76].

Interestingly, when compared with other YAP fusions, there were multiple unique features of YT that differentiate it from other YAP fusions, or a hyperfunctional form of YAP. While YT significantly upregulated YAP/TEAD transcriptional genes, RCAS tumors driven by YT formed a unique transcriptional cluster compared with tumors driven by other YAP fusions. They further demonstrated that, while all YAP fusions and a hyperfunctional form of YAP (YAP S127A/S397A) bind to TEAD1-, TEAD2-, TEAD4-, BATF-, and RUNX-specific genomic sites, YT binds to a unique set of consensus sequences. Specifically, YT binds to the TFE3, MITF, and SP1 genomic binding sites, which are normally occupied by the wild-type TFE3 protein [79]. Mechanistically, the C-terminus of TFE3 contains a basic helix–loop–helix/leucine-zipper domain, which mediates DNA binding through dimerization. As the YT fusion contains both a TEAD-binding motif from the YAP portion and a basic helix–loop–helix domain retained from the TFE3 portion, the YT transcriptional and genomic binding landscape is unique from other YAP fusions (Figure 2). The unique transcriptional and binding profile observed in YT tumors results in phenotypic alterations. YT-driven tumors displayed a unique histologic morphology that was not observed in the other YAP fusions [76].

In a study published the following year, Merritt et al. (2021) aimed to identify the mechanism of action of YT fusion [77]. This study demonstrated that the tumorigenic capacity of YT fusion was achieved via its ability to transform NIH3T3 and SW872 (liposarcoma) cells. This group was further able to recapitulate the findings of Szulzewsky et al., who found that YT induces a transcriptional profile that overlaps with both an overactive form of YAP (YAP5SA) and the wild-type TFE3. They also demonstrated that YT requires TEAD binding for its tumorigenic phenotype. Finally, they demonstrated that the genic loci occupied by YT were determined by contributions from both the YAP and TFE3 portions of the fusion protein.

Overall, these two studies have elucidated the significant role played by the TFE3 portion of the fusion protein, as it alters the DNA-binding profile, transcriptional landscape, and phenotype of the resultant tumors. Therefore, considering this fusion to be an overactive form of YAP is an oversimplification [80].

## 6. C-Terminal Proteins Reshape the Chromatin Structure and Enhance Tumorigenesis

While the functions of the N-terminal TAZ and YAP portions present in YAP/TAZ fusions have been extensively researched, only recently has our understanding of the functional contribution of the C-terminal portion of these fusions expanded. Merritt et al. aimed to elucidate how the C-terminuses (CAMTA1 in TAZ–CAMTA1 and TFE3 in YAP–TFE3) contribute to the tumorigenicity of the fusion proteins [77]. Initially, they demonstrated that the cells transformed with TC and YT displayed much greater chromatin accessibility at the promoter and enhancer elements than cells transformed with hyperactive TAZS4A and YAPS5A, respectively. Furthermore, these changes closely mirrored a significant increase in the chromatin accessibility when cells were transfected with either CAMTA1 or TFE3.

They further identified that these fusion proteins interact with a unique set of chromatin-remodeling proteins that are not utilized by TAZ or YAP. These include members of the SWI/SNF-, NUA4/TIP60-, and COMPASS-like complexes, as well as other monomeric transcriptional regulators and histone modifiers. One of the most intriguing clusters of interactors is the Ada-Two-A-containing complex (ATAC) chromatin remodeler. Specifically, ATAC is a multimeric protein complex that contains one of two histone acetyltransferases: KAT2A, or its paralog, KAT2B, as well as an additional KAT14 acetyltransferase subunit [81]. While KAT2A and KAT2B mainly acetylate H3K9 and H3K14 residues, the KAT14 subunit acetylates at the H4K16 position [81,82,83]. Whereas H3K9Ac and H3K14Ac modifications are associated with transcriptional activation, the H4K16Ac modification is associated with both transcriptional activation and repression [84,85]. In addition to KAT14′s acetyltransferase activity, the protein is important for the assembly of a functional ATAC complex, and the loss of this protein causes early embryonic lethality [86]. The activity of the ATAC complex has also been associated with non-small cell lung cancer tumorigenesis, with the *YEATS2* subunit being highly amplified in clinical non-small cell lung cancer samples [87]. In Merritt et al., a targeted RNAi screen showed that the knockdown of ATAC complex subunits inhibited the anchorage-independent growth in cells transformed with TC and YT, thereby demonstrating that the association of these oncoproteins with these chromatin remodelers is required for tumorigenesis.

## 7. Novel Model Systems for Studying EHE Biology

Two recent studies have generated genetically engineered mouse models of EHE, which conclusively demonstrated that TC is sufficient to generate EHE in vivo. In our study, Seavey et al. generated a conditional knock-in mouse model in which the wild-type *Wwtr1* locus is converted into a *Wwtr1–Camta1* locus through the utilization of a flip-excision cassette and Cre-recombinase [88]. As the *Wwtr1–Camta1* transcript remains under the control of the *Wwtr1* promoter element, the epigenetic control mechanisms of the transgene are maintained. Tumors were observed in mice when TC fusion was induced using either a ubiquitously expressed Cre, *Rosa26-CreERT2*, or an endothelial-specific Cre, the *Cdh5–CreERT2* driver. These tumors most commonly developed along the surface of the diaphragm and involved retroperitoneal structures, such as the kidney and pancreas. Lung, liver, and soft-tissue tumors were similarly identified, but at a lower frequency. These tumors were histological mimics of human EHE and expressed key EHE and endothelial markers, which were verified using IHC. To validate these tumors as replicas of the human EHE, we performed a whole-transcriptomic analysis of human EHE, as well as differential-expression analyses of other endothelial cell tumors and control tissues, and identified a gene set of 93 transcripts that were enriched in human EHE. Tumors from our mouse model showed significant enrichment of the human EHE gene set. Finally, using gene-set-enrichment analysis, we demonstrated that both human EHE and murine EHE tumor cells were enriched in YAP/TAZ transcriptional target genes.

This work left multiple unanswered questions regarding the origins of EHE. The first question is why two copies of the *Wwtr1–Camta1* allele are necessary for the generation of tumors. This could be explained either by a gene dosage effect, whereby increased levels of the *Wwtr1–Camta1* transcript are necessary to generate tumors in mice, or by a dominant-negative effect, where wild-type TAZ functionally suppresses the action of TC in vivo and inhibits tumorigenesis. We also found it particularly interesting that the ubiquitous conversion of the *Wwtr1* locus to a *Wwtr1–Camta1* locus by the expression of Cre from the ubiquitously expressed Rosa26 locus solely produced EHE. As TAZ is commonly dysregulated in cancer, one might expect that the expression of a hyperfunctional form of TAZ may generate multiple different cancer types; however, this phenotype was not observed. We hypothesized that this was due to the high transcriptional activity of *Wwtr1* within endothelium, as endothelial cells constitute the top *Wwtr1*-expressing cells within mice by single-cell RNA sequencing. While endothelial cells, or their precursors, are the cells of origin for EHE, it is uncertain whether these tumors originate from a particular endothelial-cell subtype. In addition to the mature endothelium, there are multiple types of endothelial progenitor cells and trilineage (which gives rise to venous, arterial, and lymphatic) endothelial stem cells, which are necessary for the maintenance of the vascular system [89,90,91]. In our study, we demonstrated that EHE displays a stem-cell-like phenotype; however, it has not been demonstrated conclusively whether this phenotype is a remnant of the cell of origin from which the tumor cell originates, or whether cellular transformation from normal to cancer leads to dedifferentiation.

In a different study, Driskill et al. generated an overexpression system for TC expression based on a Tet-Off approach [92]. Concordant with the findings of Seavey et al., this study demonstrated that TC expression is lethal during embryogenesis. Upon the postnatal withdrawal of doxycycline, these mice developed hyperplastic lesions within the lung vasculature. Single-cell RNA sequencing revealed that cells from these lesions were enriched in YAP/TAZ target genes when compared with other pulmonary endothelial cells. They further demonstrated that following P40, when histologically apparent tumors would be visible, the loss of TAZ–CAMTA1 expression via treatment with doxycycline led to significant lesion involution. Finally, the coexpression of TAZ–CAMTA1 and dominant-negative TEAD completely inhibited tumor formation, which suggests that TEAD activity is important for transformation. These two studies represent significant advancements in EHE research and offer new methods to investigate the biology of this disease.

## 8. Development of Targeted Therapies

**Figure 3:**
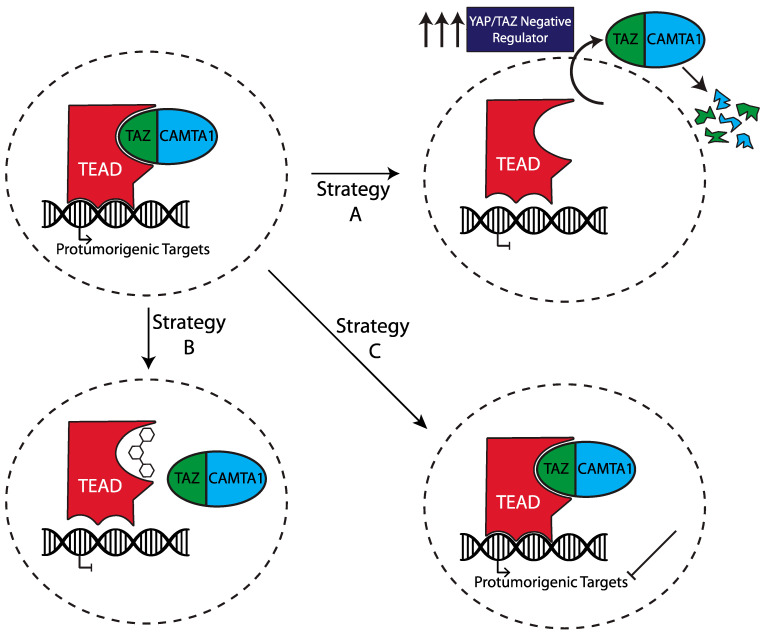
Strategies for targeting the oncogenic effects of TAZ–CAMTA1. Top left demonstrates the normal oncogenic effect of TC. Strategy A demonstrates inhibition of TC via increasing the action of negative regulators leading to cytoplasmic retention and degradation. Strategy B demonstrates inhibition by protein–protein interaction to disrupt the interaction between TC and TEAD proteins. Strategy C demonstrates inhibition of the downstream targets of TC/TEAD transcription, which promote oncogenesis.

Similar to other monogenic cancers that contain a single-driver mutation (e.g., chronic myelogenous leukemia, gastrointestinal stromal tumor, etc.), targeting the TAZ–CAMTA1-driver oncoprotein either directly or indirectly will likely result in a profound clinical effect [93,94,95,96]. Indeed, the clinical efficacy of targeted therapies in these monogenic cancers is greater than is observed with targeted therapy against cancers with more complex genetic landscapes. Multiple avenues are being actively pursued to target EHE and its associated fusion protein. Generally, there are three different therapeutic mechanisms that are simultaneously being investigated for inhibiting the oncogenic effect of the fusion protein within EHE: (A) leverage the “Hippo-dependent” and “Hippo-independent” mechanisms upstream of the fusion to degrade the fusion or shuttle it to the cytoplasm; (B) use drugs that directly act on the TC/TEAD transcriptional complex; (C) identify and target oncogenic signaling downstream of the fusion (Figure 3) [45]. Herein, we present an overview of both proposed and currently utilized targeted therapies for EHE.

### 8.1. Leverage the “Hippo-Dependent” and “Hippo-Independent” Mechanisms Upstream of the Fusion

As wild-type YAP/TAZ are regulated both by the Hippo pathway and other signaling pathways directly, one can envision that the upstream regulation of TC may occur via both “Hippo-dependent” and “Hippo-independent” mechanisms. An unresolved controversy is whether the TC protein is regulated by upstream controls that normally regulate YAP/TAZ. If the TC fusion protein is controlled by these regulators, the action of TC could be diminished, either by the inhibition of the negative regulators of the Hippo pathway, or by positive regulators of TC. Despite the initial suggestion that TC is unregulated by the Hippo pathway, recent evidence suggests that Hippo alters the action of TC [92]. In unpublished work, via co-immunoprecipitation followed by mass spectrometry, we have seen that the TC protein is phosphorylated at the regulatory S89 residue and that it associates with the 14-3-3 protein [97]. This work was further corroborated by Driskill et al., who showed that TC associates with subunits of the 14-3-3 complex. They also showed both the enhanced nuclear localization of TC in LATS knockout cells compared with that in LATS wild-type cells, and the enhanced nuclear localization and transcriptional activity of the fusion protein upon the mutation of the serine residues within TC, which are phosphorylated by LATS [92]. Therefore, it is becoming more evident that, even though TC is localized predominantly to the nucleus, it is not completely dissociated from the regulation of the Hippo pathway. However, further research is necessary to understand the specific pathways that regulate TC, and to what extent.

If LATS kinase activity can restrict the nuclear localization and transcriptional activity of TC, augmenting the activation of LATS would be an optimal target to decrease TC activity. However, LATS is negatively regulated by several upstream protumorigenic signals, and it would likely be important to inhibit these signals in order to restore LATS activity in EHE. In other cancers, the inhibition of upstream negative regulators, including EGFRs, GPCRs, and VEGFRs, increases LATS activity [45]. Whether such a scenario is applicable to EHE remains unexplored and is worth investigating, as the drugs against these targets are already being used to treat other cancers. These therapies can be repurposed if they show efficacy against EHE in preclinical research and clinical trials. Sorafenib and bevacizumab, drugs that inhibit VEGF signaling, have shown efficacy in clinical trials involving patients with EHE, where they either improved progression-free survival or resulted in disease stabilization [98,99,100]. Being an endothelial-cell cancer, inhibiting VEGF activity is a rational target, but whether VEGF signaling also inhibits LATS phosphorylation will be an interesting aspect to investigate [101]. Another key target is the PI3K/AKT/mTOR pathway, which facilitates the nuclear localization of YAP/TAZ by inhibiting the Hippo signaling pathway [102]. Inhibitors that target this pathway hold promise. A retrospective case study provides a compelling rationale for administering such drugs, and disease stabilization was observed in patients with EHE who were treated with the mTOR inhibitor sirolimus [22,103]. Whether PI3K inhibitors also display similar effects remains to be determined. The mode of action of these drugs remains unclear. It is important to verify whether drugs that target the PI3K pathway act in a Hippo-dependent manner and restrict TC localization, or via an alternative mechanism.

A notable example of “Hippo-independent” TC regulation is the mevalonate-synthesis pathway, which appears to positively regulate TC activity [104]. Therefore, inhibiting mevalonate synthesis through the use of 3-hydroxy-3-methyl-glutaryl-coenzyme A reductase inhibitors, and notably simvastatin, may offer therapeutic benefits to EHE patients [105]. Isoprene precursors generated as secondary products in the de novo cholesterol-synthesis pathway are necessary for the prenylation of many regulatory proteins, including Ras-homologous GTPases (Rho) [104]. Rho kinases and the Rho pathway integrate multiple upstream signals to yield downstream responses [3,19,104]. For instance, Rho activation leads to the inhibition of YAP/TAZ, which, in turn, increases their transcriptional activity [12,104]. Driskill et al. demonstrated, in a heterologous cell-line model, that simvastatin abrogates the anchorage-independent growth of TC-transformed cells and suppresses the expression of TC transcriptional targets, suggesting that the Mevalonate/Rho axis may regulate TAZ–CAMTA1 activity [92]. This work was further supported by a recent retrospective review from the MD Anderson Cancer Center, which aimed to identify whether prognosis was affected in EHE patients who were on statins for other indications [106]. This study showed a greater median survival among patients on statin therapy than among those not on statin therapy. However, this study has limitations, as there were other significant covariates between the groups, and the sample size was too small to identify a statistically significant difference. Nevertheless, preclinical cell-line work, coupled with this retrospective study, warrants additional preclinical research that is aimed at identifying the efficacy of statins in the treatment of EHE.

Importantly, YAP–TFE3 appears to be less regulated by the Hippo pathway than TC, and diminishing the function of YAP–TFE3 via the augmentation of Hippo-pathway activity is likely not a potent therapeutic mechanism [76,77]. This is altogether unsurprising, as all identified YAP–TFE3 fusions have four out of the five LATS sites truncated, including the key S127 residue [59,60,76].

### 8.2. Use Drugs That Directly Act on the TC/TEAD Transcriptional Complex

As previously mentioned, TC is a transcriptional coactivator that interacts with the TEAD family of transcription factors and relies on TEAD activity to alter transcription. Therefore, if a small molecule can be designed to disrupt the TC–TEAD interaction, the transcriptional activity of the entire complex can be potently inhibited [45]. As TC largely mimics YAP/TAZ in its mechanism of action, TEAD inhibitors or YAP/TAZ–TEAD disruptors can be used to inhibit TC [75,88,107]. Currently, there are three phase 1 clinical trials investigating the utility of novel compounds that aim to disrupt the YAP/TAZ–TEAD complex. In all three of these trials, the primary disease being targeted is NF2-mutant mesothelioma and other NF2-mutant solid tumors. As NF2 acts as an activator of MST1/2 phosphorylation, NF2 loss leads to the inhibition of the Hippo pathway and the overactivation of YAP/TAZ signaling [29]. The specific compounds used in these trials are IAG933 (NCT04857372, Novartis, Basel, Switzerland), IK-930 (NCT05228015, Ikena Oncology, Boston, MA, USA), and VT3989 (NCT04665206, Vivace Therapeutics, San Mateo, CA, USA) [108,109,110]. Some of these trials include the enrollment of EHE patients, and so it will be interesting to see whether these drugs will have efficacy in EHE patients. 

### 8.3. Identifying and Targeting the Oncogenic Signaling Downstream of the Fusion

We recently published a study that demonstrated that TC upregulates connective tissue growth factor (CTGF), and that the expression of this protein is important for oncogenesis [111]. CTGF binds to integrins on the cell surface, and the knockdown of cell-surface integrins abrogates the anchorage-independent growth of cells that were transformed by TC. The knockdown of CTGF expression in tumor xenografts reduced the rate of tumor growth. More importantly, we observed that CTGF drives dysregulated MAPK activity, and we used a previously approved inhibitor of dual-specificity mitogen-activated protein kinase 1 and 2 (MEK1/2) in our assays, trametinib, to inhibit TC-dependent growth. Trametinib is used clinically for the treatment of metastatic and advanced-stage BRAF-mutant melanoma. As hypothesized, trametinib inhibited TC-dependent growth in vitro in colony-formation assays, and potently inhibited TC-dependent growth in vivo. This evidence led us to develop a prospective clinical trial of trametinib in patients with EHE (NCT03148275) [112]. The results of this study are forthcoming.

## 9. Conclusions

Novel model systems, which have only recently been developed, have greatly enhanced our understanding of EHE and have conclusively demonstrated that YAP/TAZ fusions drive EHE and YAP1–TFE3-fused hemangioendothelioma. These fusions function as dysregulated forms of YAP/TAZ and promote tumorigenesis. However, there are key unanswered questions regarding the cellular origin of the tumors, mechanisms of tumorigenesis, and specific dependencies that facilitate oncogenesis. However, we are poised to use these recently developed and faithful EHE models to answer these questions and many more.

TC fusion is the only observed abnormality in roughly 50% of the EHE cases, which makes EHE a “genetically clean” model system to understand YAP/TAZ-mediated oncogenesis. With a growing body of evidence demonstrating the central role of YAP/TAZ dysregulation in promoting tumorigenesis, and especially aggressive tumor phenotypes, targeted therapies that inhibit their functions may yield significant therapeutic efficacy. While these therapeutics are in the early stages of testing, the identification of cancers most likely to respond to these therapies is necessary for adequate clinical-trial design. As EHE samples universally express an activating fusion containing TAZ (most commonly TAZ–CAMTA1), EHE presents a robust system for evaluating these therapies.
